# Outer membrane vesicle-mediated release of cytolethal distending toxin (CDT) from *Campylobacter jejuni*

**DOI:** 10.1186/1471-2180-9-220

**Published:** 2009-10-16

**Authors:** Barbro Lindmark, Pramod Kumar Rompikuntal, Karolis Vaitkevicius, Tianyan Song, Yoshimitsu Mizunoe, Bernt Eric Uhlin, Patricia Guerry, Sun Nyunt Wai

**Affiliations:** 1 Department of Molecular Biology, Umeå University, S-90187 Umeå, Sweden; 2 Department of Bacteriology, the Jikei University School of Medicine, Tokyo 105-8461, Japan; 3 Laboratory for Molecular Infection Medicine Sweden (MIMS), Umeå University, S-90187 Umeå, Sweden; 4 Enteric Diseases Department, Naval Medical Research Center, 503 Robert Grant Ave, Silver Spring, MD, USA

## Abstract

**Background:**

Background: Cytolethal distending toxin (CDT) is one of the well-characterized virulence factors of *Campylobacter jejuni*, but it is unknown how CDT becomes surface-exposed or is released from the bacterium to the surrounding environment.

**Results:**

Our data suggest that CDT is secreted to the bacterial culture supernatant via outer membrane vesicles (OMVs) released from the bacteria. All three subunits (the CdtA, CdtB, and CdtC proteins) were detected by immunogold labeling and electron microscopy of OMVs. Subcellular fractionation of the bacteria indicated that, apart from the majority of CDT detected in the cytoplasmic compartment, appreciable amounts (20-50%) of the cellular pool of CDT proteins were present in the periplasmic compartment. In the bacterial culture supernatant, we found that a majority of the extracellular CDT was tightly associated with the OMVs. Isolated OMVs could exert the cell distending effects typical of CDT on a human intestinal cell line, indicating that CDT is present there in a biologically active form.

**Conclusion:**

Our results strongly suggest that the release of outer membrane vesicles is functioning as a route of *C. jejuni *to deliver all the subunits of CDT toxin (CdtA, CdtB, and CdtC) to the surrounding environment, including infected host tissue.

## Background

*Campylobacter jejuni *is recognized as the most frequently isolated bacterial cause of food-borne gastroenteritis worldwide. It is found in both developed and developing parts of the world [1,2]. Clinical illness ranges from mild self-limiting, non-inflammatory diarrhea to severe inflammatory bloody diarrhoea that may be associated with pyrexia and bacteriaemia [1]. In addition, *Campylobacter enteritis *has been associated with subsequent development of Guillain Barré syndrome, an acute inflammatory polyneuropathy [3]. Although various virulence factors such as adherence and invasive abilities and toxin production and motility have been implicated [4-8], the precise mechanism(s) involved in the pathogenesis is yet to be elucidated. The pathogenesis of *C. jejuni *is poorly understood, partly because of the lack of a suitable animal model and partly due to the difficulties in genetic manipulation [9]. Bacterial toxins have been considered important factors for the pathogenesis of Campylobacter infection. The best characterized toxin of *Campylobacter *spp. is the cytolethal distending toxin (CDT). The *C. jejuni cdt *operon consists of three adjacent genes, *cdtA*, *cdtB *and *cdtC*, that encode proteins with predicted molecular masses of 27, 29 and 20 kDa, respectively [10]. The effect of CDT was first described as an activity in culture supernatants of *Campylobacter *spp. and of certain enteropathogenic strains of *Escherichia coli *that caused eukaryotic cells to slowly distend over a period of 2-5 days, eventually leading to cell death [11]. CDT appears to be common in *C. jejuni *strains *e.g*. in one study of 117 isolates there was positive evidence for CDT in 114 of the isolates in Vero cell assays [12]. A study in Bahrain showed that among the 96 *C. jejuni *strains examined, 80 (83.0%) were *cdtB *positive and 16 (17.0%) were negative by PCR [13]. Recently, Jain et al described that the presence of the *cdtB *gene in *C. jejuni *was associated with increased adherence to, invasion of and cytotoxicity towards HeLa cells [14]. The significant pathological changes in the colons of mice treated with the supernatant containing *C. jejuni *CDT suggested that CDT is an important virulence attribute and that the colon is the major target of CDT.

CDT belongs to a family of bacterial protein toxins that affects the epithelial cell layer and interrupts the cell division process with resulting cell cycle arrest and cell death [10,15]. CDT activity is not unique to *E. coli *and *Campylobacter *spp. but has been described in various other Gram-negative bacteria including *Shigella *spp., *Helicobacter hepaticus*, *Haemophilus ducreyi*, and *Actinobacillus actinomycetemcomitans*. [16]. It has been suggested that CDT is a tripartite "AB_2_" toxin in which CdtB is the active toxic unit; CdtA and CdtC make up the "B_2_" units required for CDT binding to target cells and for delivery of CdtB into the cell interior [17]. The primary effect of the CDTs, regardless of their bacterial origin, is eukaryotic cell cycle arrest at the G2/M stage with resultant cessation of cell division due to the DNase activity of the CdtB subunit [4,17,18].

Interestingly, in the case of *Salmonella typhi*, that is lacking the genes for CdtA and CdtC, the CdtB protein was delivered into the target cell upon entry of this invasive bacterium [19]. It was proposed that *S. typhi *synthesizes and secretes CdtB once it has reached an intracellular compartment of the host cell where the toxin can be either retrotranslocated to the cytosol or transported to a compartment where retrotranslocation can take place. Three subunits of CDT appear to be constitutively synthesized, assembled into a CDT complex and translocated into the periplasm in bacterial cells [20] The CDT complex is then secreted into the culture supernatant, probably via CdtA that undergoes post-translational cleavage at its N-terminal signal sequence [20,21]. It has been shown that a proper complex of CdtA, CdtB and CdtC and its binding to the host cell are required for maximal cytotoxic activity [22]. In case of CDT from *Actinobacillus actinomycetemcomitans*, upon binding of the holotoxin to the target cells, CdtB is internalized whereas CdtA and CdtC likely remain associated with the membrane [23]. In *S. typhimurium *it was described that the CdtB protein has a Sec-dependent secretion signal sequence at the amino terminal end that is cleaved during translocation of the protein across the cytoplasmic membrane into the periplasmic space where CdtB undergoes folding and assembly to form the mature protein. A *S. typhi *mutant lacking the Sec-dependent signal sequence for CdtB was not cytotoxic [19]. However, it has remained unclear how CDT becomes surface-exposed and released from the different bacterial cells.

In general, proteins have to reach their final destination to exhibit their physiological functions. Outer membrane vesicles (OMVs) are common to a wide variety of Gram-negative bacteria and are produced during the course of normal metabolism and cell growth. As OMVs are blebs from the outer membrane, the outer membrane associated protein(s) as well as some periplasmic components are released in association with OMVs. Once the OMVs are free from the bacterium, they appear as small membrane vessels including periplasmic constituents and outer membrane components. The role of OMVs is likely multifaceted: OMVs may act as delivery vehicles for bacterial toxins lacking typical signal sequences [24-28], promote cell-cell communication via transit of signalling molecules [29], and can inhibit phagosome-lysosome fusion during macrophage infection [30]. OMVs are potentially rich in antigens that serve as initial targets for innate and adaptive immune recognition [31], generating protective immunity against bacterial challenge when used as an immunogen [32]. Ricci *et al*. found that a portion of secreted VacA toxin from *H. pylori *was OMV-associated and that the OMV-associated VacA caused a statistically significant vacuolation of gastric epithelial cells [33]. They therefore suggested that OMVs may represent an important vehicle for delivering virulence factors to the gastric mucosa and that OMV-associated VacA could play a pathobiological role different from that of free and soluble toxin. There is no detailed study of OMVs from *C. jejuni*. Here we report that biologically active CDT is secreted from *C. jejuni *bacterial cells in association with OMVs.

## Methods

### Bacterial strains and culture conditions

*C. jejuni *strain 81-176 [34,35] and its mutant derivative DS104 *cdtA*::km [20] were used in our experiments. *C. jejuni *strains were grown on Mueller-Hinton agar plates supplemented with kanamycin (Km 25 μg/ml) when needed, under microaerobic conditions at 42°C.

### Cell line media and culture conditions

The human ileocecum carcinoma cell line HCT8 (ATCC number CCL-224) was kindly provided by the Institute for Molecular Infection Biology, University of Würzburg. HCT8 cells were cultured in RPMI 1640 (Gibco) supplemented with 2 mM glutamine, 1 mM pyruvate, 10% FCS and 50 μg/ml gentamicin. The cells were cultivated at 37°C in a 5% CO_2 _atmosphere.

### Isolation of outer membrane vesicles

OMVs were isolated from culture fluid as previous described [25] with some modifications. Briefly, bacteria were inoculated in a 600 ml tissue culture flask containing Muller-Hinton agar and 100 ml of Muller-Hinton broth (biphasic media) and incubated under microaerobic conditions for 24 h. Bacterial cells were removed from culture fluid by centrifugation at 5000 × *g *for 30 min. The supernatants were filtered through a 0,45 μm-pore-size membrane filter (Sartorius). The cell-free supernatants were centrifuged at 100 000 × *g *for 2 h at 4°C in a 45 Ti rotor (Beckman Instruments Inc.) to pellet the vesicles. The vesicles were suspended in 20 mM Tris-HCl (pH 8.0) or 50 mM HEPES. The proteins in the supernatants collected before and after OMV isolation, respectively, were concentrated by trichloroacetic acid precipitation.

### Atomic force microscopy

Ten μl of the vesicle samples were placed onto freshly cleaved mica (Goodfellow Cambridge Ltd., Cambridge, United Kingdom). The samples were blot dried and desiccated prior to imaging. Imaging was done on a Nanoscope IIIa (Digital Instruments, Santa Barbara) Atomic Force Microscope using Tapping ModeTM. A silicon probe was oscillated at its resonant frequency of approximately 300 kHz, selected by the Nanoscope software. Images were collected in air at a scan rate of 0.8-1.5 Hz, depending on scan size and sample number (512 or 256 samples/image). The final images were plane fitted in both axes and presented in a surface plot of the height mode.

### Cell fractionation

For the whole cell lysate fractions, the bacteria (100 μl) from the cultures were centrifuged at 12,000 × g for 5 min and 5 μl bacterial suspensions were loaded in the well. The bacteria (1 ml samples from cultures with a cell density of ca 5 × 10^9^/ml) were harvested by centrifugation and washed twice in a 0.2 volume of ice-cold 0.01 M Tris-HCl (pH 8.0), 0.3 M NaCl before being resuspended in 0.2 ml of 0.03 M Tris-HCl (pH 8.0), 20% (wt/vol) sucrose, and 0.1 mM EDTA at 25°C. After 10 min the cells were pelleted and rapidly suspended in 0.3 ml of ice-cold 0.5 mM MgCl_2_. After incubation on ice for 10 min, the cells were removed by centrifugation at 12,000 × *g*. The supernatant was used as the periplasmic protein extract. The cell pellet was then disrupted by sonication in 0.5 ml ice-cold water. The cell debris and unbroken cells were removed by centrifugation at 5,000 × *g *for 10 min at 4°C, and the next supernatant was fractionated into the membrane and cytoplasmic fractions by centrifugation at 10,000 × *g *for 30 min at 4°C. The resulting supernatant was used as a cytoplasmic fraction. The sediment was resuspended in sterile distilled water and used as the membrane fraction. In order to separate the inner and outer membranes, the membrane fraction was further treated with *N*-lauryl sarcosyl at a final concentration of 2% at room temperature and then centrifuged at 15,000 × *g *for 30 min. The resulting sediment was used as the outer membrane fraction, and the supernatant was used as the inner membrane fraction after dialysis and precipitation. Extracellular, periplasmic, cytoplasmic, and membrane-bound proteins were concentrated by precipitation with ice-cold trichloroacetic acid (final concentration, 10%). The precipitated proteins were collected by centrifugation at 12,000 × *g*, washed with acetone, dried under vacuum, and dissolved in sample buffer (50 mM Tris-HCl [pH 6.8], 10% glycerol, 5% β-mercaptoethanol, 2% sodium dodecyl sulfate [SDS], 0.05% bromophenol blue). Samples were neutralized by addition of a saturated Tris solution and boiled for 5 min at 100°C before SDS-PAGE analysis. The amount of sample from each extract used for the SDS-PAGE was as follows: 2.5 μl of the 150 μl cytoplasmic (C) extract; 2.5 μl of the 40 μl inner membrane (IM) extract; 5 μl of the 100 μl periplasmic (P) extract; 2.5 μl of the 40 μl outer membrane (OM) extract and 2.5 μl of the 300 μl whole cell (WC) extract. In all cases the extracts were derived from 1 ml culture samples and the relative amount of the extracts used for SDS-PAGE in comparison with the amount of WC extract used (arbitrarily set to 1.0) were 2 × for C; 8 × for IM; 6 × for P, and 8 × for OM.

### SDS-PAGE and N-terminal sequencing

SDS-PAGE was performed using the method described by Laemmli [36]. Proteins were blotted onto PVDF membrane and stained with Coomassie brilliant blue. 50% methanol was used for de-staining the membrane to visualize the protein bands. Proteins present in visible bands were excised from the membrane for N-terminal sequencing. Determination of the N-terminal amino acid sequence of proteins was achieved by automated Edman degradations of samples blotted onto PVDF membranes. The sequencing was performed on a Procise 494 Sequencer (Applied Biosystems) with an on-line PTH-analyzer. A sufficient number of residues were determined to allow unambiguous identification (100% identity; using the NCBI BLAST database). PageRuler Prestained Protein Ladder #SM0671 marker (Fermentas) and low range molecular weight markers RPN 755 (Amersham Biosciences) were used as molecular weight markers of proteins and LPS in the SDS-PAGE silver stained gels.

### Western immunoblot analysis

The isolated vesicles and the different sub-cellular extracts (see below) were subjected to polyacrylamide gel electrophoresis and then blotted onto a PVDF membrane. Proteins were identified using different primary polyclonal antisera at a final dilution of 1:5000 against CdtA, CdtB, CdtC [20], an anti-Omp50 antiserum at a final dilution of 1:5000 [37], an anti-HtrA (*E. coli*) antiserum at a final dilution of 1:7500 [38], and anti-CRP antiserum at a final dilution of 1:3000 [39]. For CRP detection, we used *E. coli *anti-CRP antiserum since the CRP proteins from *C. jejuni *and *E. coli *have 80% identity at protein level. Anti-rabbit horseradish peroxidase-conjugate was used as a secondary antiserum at a final dilution of 1:20,000. The ECL^+ ^chemiluminescence system was used to detect the level of chemiluminescence that was then monitored using a Flour-S MultiImager (BioRad) and by autoradiography.

### Lipooligosaccharide analysis and staining

Lipooligosaccharide (LOS) samples were prepared from whole-cell lysates (0.1 ml samples) and OMVs (50 μl samples of the OMV preparations). The samples were subjected to complete digestion with proteinase K as described earlier [40]. The isolated LOS samples (2.5 μl of the whole cell extracts and 10 μl of the OMV extracts, respectively) were separated on 16% Tricine gels (Invitrogen, Carlsbad, CA, USA) and then silver stained [41].

### Dissociation assay

Vesicle samples (60 μg/ml total protein) in 50 mM HEPES (pH 7.3) were incubated on ice for 1 hour in the absence or presence of either NaCl (1 M), Na_2_CO_3 _(0.1 M) pH 10.0, Urea (8 M) or 1% SDS [28]. Samples were then centrifuged at 100,000 × *g *for 2 hours at 4°C and both pellet and supernatant fractions were analyzed by SDS-PAGE and immunoblot analyses using anti-CdtA, anti-CdtB, anti-CdtC polyclonal antiserum and anti-GroEL polyclonal antiserum against *E. coli *GroEL protein. Before loading, the soluble proteins in the supernatant were concentrated by TCA-precipitation.

### Electron microscopy and immunogold labeling

Samples from vesicle preparations were negatively stained with a solution of 0.1% uranyl acetate on carbon coated Formvar grids and examined under the electron microscope. Micrographs were taken with a JEOL 2000EX electron microscope (JEOL Co., Ltd., Akishima, Japan) operated at an accelerating voltage of 100 kV.

For immunoelectron microscopy, a colloidal gold probe (Wako Pure Chemical Industries Ltd., Osaka, Japan) was used to label the specific reaction sites of anti-CDT sera in the specimens of OMVs from *C. jejuni*. To label the specimens, a 50 μl sample (ca 3 μg protein) of the OMV preparation was treated with antiserum appropriately diluted in phosphate-buffered saline (PBS) for 30 min at 37°C. The OMVs then were separated from the serum by centrifugation at 100,000 × *g *for 2 h at 4°C. After being washed three times with PBS, the OMV samples were mixed with a suspension of the colloidal gold probe, and the mixture was kept at room temperature for 30 min. After being washed with PBS to remove unbound gold particles, the OMV samples were negatively stained with 0.1% uranyl acetate on carbon coated Formvar grids and examined under the electron microscope.

### Cytolethal distending assays with HCT8 cells

HCT8 cells were seeded in 24-well plates (Falcon) and grown to 50% confluence. 50 μl of vesicle samples (ca 3 μg protein) were added to the cells. The occurrence of cytotoxic effects was monitored for up to 72 h. Cells were fixed with 2% paraformaldehyde in PBS pH 7.3 for 10 min. After fixation, cells were washed twice with PBS and incubated with 0.1 M glycine for 5 min at room temperature. After washing twice with PBS, the cells were permeabilized with 0.5% Triton X-100 (Sigma-Aldrich). Actin was stained with Alexa Fluor 488 phalloidin (Molecular probes, Invitrogen, Oregon, USA) containing 1% BSA (Sigma-Aldrich). After thorough washing with PBS, the nuclei were stained with DAPI (Sigma-Aldrich) (1:5,000) for 5 min before mounting in Mowiol (Scharlau Chemie S. A.) containing antifade (P-phenylene diamine). Cells were analysed using a Zeiss Axioskop routine microscope and photographed with a Hamamatsu digital camera.

### Thymidine incorporation analysis

DNA synthesis was assessed by measuring [^3^H] thymidine incorporation in HCT8 cells. Cells were seeded in 96-well plates and grown to 25% confluence. After 48 h of incubation with 10 μl of OMVs (0.6 μg protein) from strains 81-176 and its *cdtA*::km mutant, [^3^H] thymidine (0.5 μCi/well; Amersham) was added and the incubation was continued for another 4 h. Cells were harvested with a SKATRON semiautomatic cell harvester and [^3^H] thymidine uptake was determined with a Beta Counter (LKB Wallace 1218 Rackbeta liquid scintillation counter).

## Results and discussion

### Analyses of OMVs from *C. jejuni*

**Figure 1:**
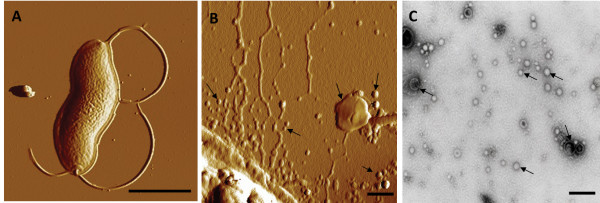
**Surface structure analyses of *C. jejuni***. Atomic force micrographs of (A) a *C. jejuni strain *81-176 cell (Bar: 1 μm) and of (B) small and large OMVs (examples indicated by arrows) on the surface of a *C. jeuni *cell (Bar: 100 nm). (C) Electron micrograph of OMVs (examples indicated by arrows) isolated from *C. jejuni strain *81-176 (Bar: 100 nm).

**Figure 2:**
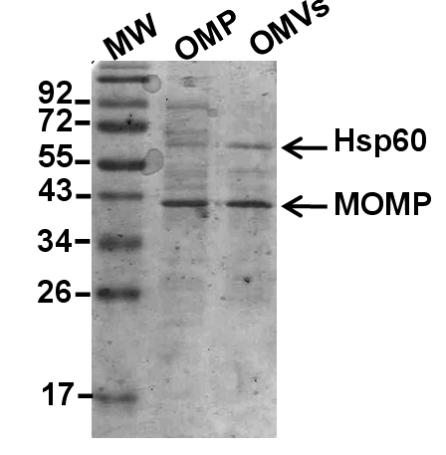
**Protein profile of *C. jejuni *outer membrane and OMVs**. Comparison of protein composition between the outer membrane protein fraction (OMP) and the OMVs sample from wild type *C. jejuni *strain 81-176. Protein bands were visualized by Coomassie blue staining of a SDS-PAGE gel.

In order to analyze the surface structure of wild type *C. jejuni *strain 81-176, we examined the bacteria by atomic force microscopy, which revealed that there were OMVs surrounding the bacterial cells (Figure 1A&1B). Since recent studies [25-28] suggest that some bacterial protein toxins are secreted in association with OMVs, we decided to determine whether CDT could be detected in association with such vesicles. We isolated the OMVs from cell-free supernatants of *C. jejuni *after growth in biphasic medium as described in Materials and Methods. Studies of the OMV samples using electron microscopy revealed that the OMVs from *C. jejuni *strain 81-176 were somewhat heterogeneous in size with a diameter in the range of 10-50 nm (Figure 1C). In order to visualize the protein components of OMVs we performed SDS-PAGE analysis. Comparison of OMV and outer membrane fraction of *C. jejuni *strain 81-176 showed that there was clear similarity of the major protein bands and most of the minor bands (Figure 2) The N-terminal amino acid sequence of the major protein band was determined. The result (N-terminal: AS/GKEIIFS) corresponding to the most abundant band at 45 kDa identified it as a major outer membrane protein (MOMP CJJ81176_1275). The presence of MOMP verified that the isolated OMVs fraction was derived from the outer membrane compartment of the bacteria. Another rather abundant protein in the OMVs fraction was found to correspond to the Hsp60 (heat shock protein 60 CJJ81176_1234). The *C. jejuni *Hsp60 protein is similar to, and may be regarded as a paralog to, GroEL proteins of *E. coli *and many other bacteria. Generally the GroEL heat shock protein is described as a cytoplasmic protein. However, there is increasing evidence of cell surface localization of GroEL from studies of different bacterial species, *e.g*. in the case of *H. pylori*, *S. typhimurium*, and *Hemophilus influenzae *[18,42,43].

### Detection of CDT proteins in association with OMVs

**Figure 3:**
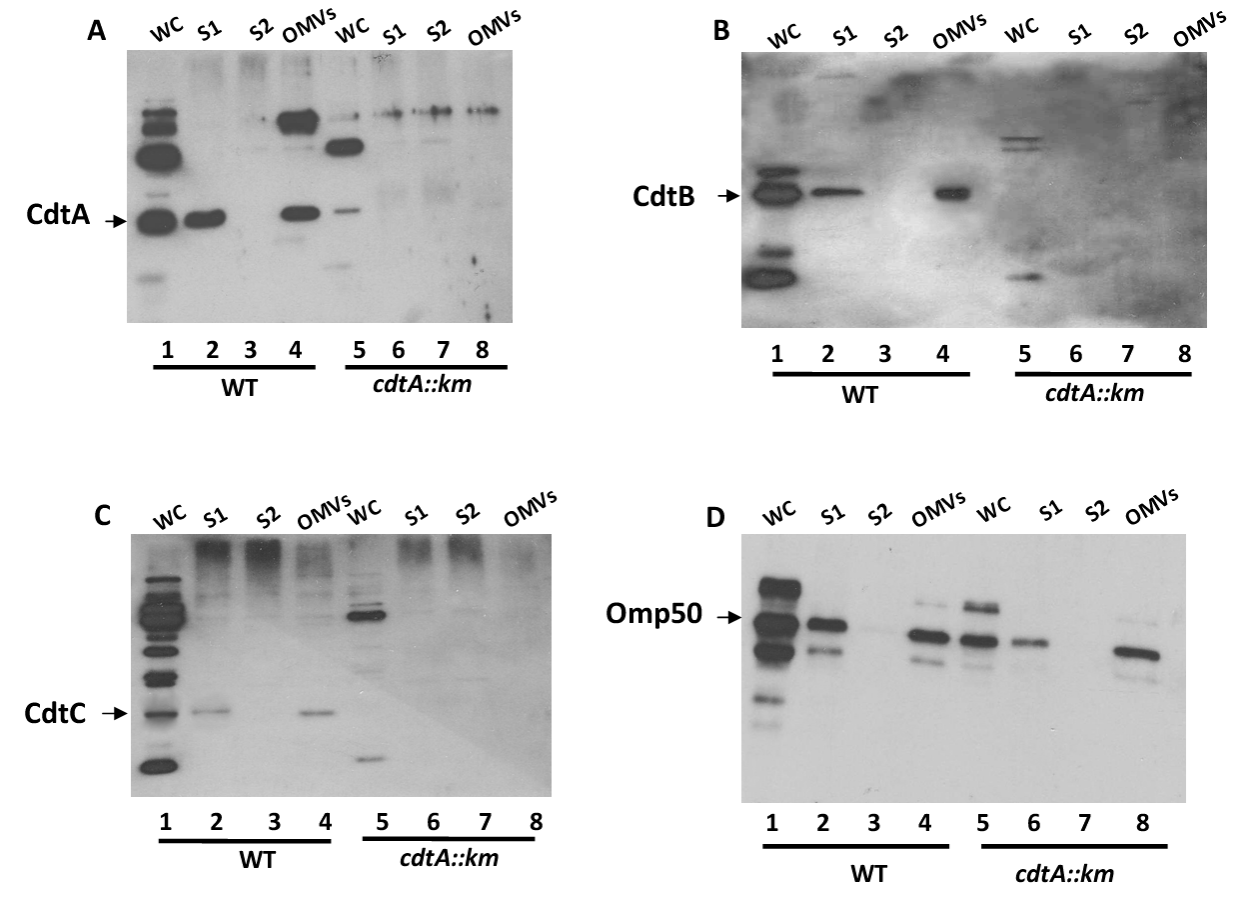
**Immunoblot detection of intra- and extra-cellular CDT of *C. jejuni***. Immunoblot analyses of samples from *C. jejuni *wild type strains 81-176 (lanes 1-4) and the *cdtA*::km mutant (lanes 5-8). Samples: 1&5; whole cells (WC), 2&6; supernatants 1 (S1), 3&7; supernatants 2(S2), 4&8; OMVs, (A) Immunoblot detection with anti-CdtA polyclonal antiserum, (B) immunodetection with anti-CdtB polyclonal antiserum. (C) immunoblot detection with anti-CdtC polyclonal antiserum. (D) immunoblot detection with anti-Omp50 polyclonal antiserum.

In order to determine whether all or a subset of the proteins constituting CDT were present in the OMVs, Western immunoblot analyses with anti-CdtA, anti-CdtB, and anti-CdtC polyclonal antisera were performed. A *cdtA*::km derivative (DS104) was used as a negative control. The insertion of the kanamycin resistance determinant has been shown to be polar on the other genes [20] in the *cdtABC *operon and none of the CDT proteins were detected in the *cdtA*::km mutant (Figure 3A-C, lanes 5-8). OMV preparations from the wild type strain were indeed associated with the CdtA, CdtB, and CdtC proteins as determined by the immunoblot analyses. The protein loading in the SDS-PAGE gel was normalized such that a total of 3 μg protein was loaded in each well. As shown in Figure 3A-C (Lane 4), all subunits could be detected in association with OMVs from the wild type bacteria. In order to rule out contamination from the cytoplasmic fraction of the bacterial cells, the OMV samples were analyzed using antiserum against the cAMP receptor protein (CRP) as a cytoplasmic marker. There was no reactive band detected with anti-CRP antiserum when supernatants and OMVs were tested (data not shown). Using an antiserum raised against the outer membrane protein Omp50 we further confirmed that the OMV fraction was derived from the outer membrane compartment of the bacteria (Figure 3D). The OMVs were also studied with regard to lipooligosaccharide (LOS) patterns using SDS-PAGE and silver staining of preparations treated with Proteinase K. The LOS was detected in the OMV samples and the pattern was identical to that of the whole cell samples (data not shown). The relative intensity of the major bands indicated that the LOS in the OMVs represented ca 0.2-0.5% of the total LOS of whole bacterial cells.

### Immunoelectron microscopic analysis of proteis in OMVs

**Figure 4:**
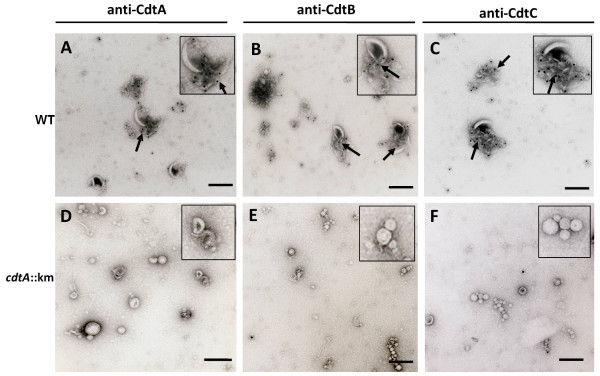
**Electron microscopy and immunogold labelling of CDT**. Immunoelectron microscopic analyses of OMVs from wild type *C. jejuni *strain. 81-176 (A-C) and the *cdtA::km *mutant (D-F) using anti-CdtA (A, D), anti-CdtB (B, E), and anti-CdtC antisera (C, F). Arrows show the gold particles associated with OMVs. The square in the upper right corners show enlargements of parts of the micrographs. Bars correspond to 100 nm.

**Figure 5:**
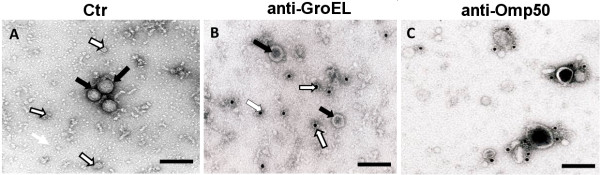
**Electron microscopy and immunogold labelling of Hsp and Omp50**. Immunoelectron microscopic analyses of OMVs. (A) OMVs of wild type *C. jejuni *strain 81-176 without antiserum (control). (B), immunolabelling with anti-Hsp antiserum. (C) immunolabelling with anti-Omp50 antiserum. White arrows show the GroEL like particles (in A) and the localization of gold particles on the GroEL like particles (in B). Black arrows show the OMVs (in A&B). Bars correspond to 100 nm.

To more directly monitor the association of CDT proteins with OMVs, we performed immunoelectron microscopic analyses. By immunolocalization using anti-CdtA, anti-CdtB, and anti-CdtC antibodies in the immunogold labeling method we detected the deposition of gold particles on the vesicles obtained from CDT-producing bacteria (Figure 4A-C), whereas there was no labeling of OMVs from the CDT-negative strain (Figure 4D-F). We observed that some CDT containing vesicles were ruptured when the OMVs samples were mixed with antiserum in the immunogold experiment. The gold particles were mainly observed on the material of the ruptured vesicles. It appeared that due to the rupture of the OMVs some of the released CDT subunits were accessible to the antiserum. The results strongly support the suggestion that the CDT proteins were indeed associated with OMVs of *C. jejuni *strain 81-176 and it appeared that the proteins might be internal or integral to the vesicle membrane. Since the *C. jejuni *Hsp60 protein that was somehow associated with OMVs as detected by SDS-PAGE analysis after the ultrcentrifugation step we also performed the immnunogold labelling and electron microscopic examination using an Hsp60 recognizing polyclonal antiserum raised against the *E. coli *GroEL protein (Sigma-Aldrish). As shown in Figure 5B the gold particles labelled with anti-Hsp60 antiserum were observed not in direct association with OMVs but gold particles were associated with some amorphous material outside the OMVs. A similar immunogold labelling and analysis of the OMVs preparation with anti-Omp50 antiserum was shown in Figure 5C. In this case the gold particles were found to be localized in direct association with the OMVs as expected for an outer membrane protein. The results from these analyses indicated that the Hsp60 protein of *C. jejuni *was not directly in association with OMVs. We suggest that the presence of GroEL in the OMVs preparation might be due merely to the co-precipitation during the vesicle isolation procedure.

### Sub-cellular localization of CDT proteins in *C. jejuni *cells

**Figure 6:**
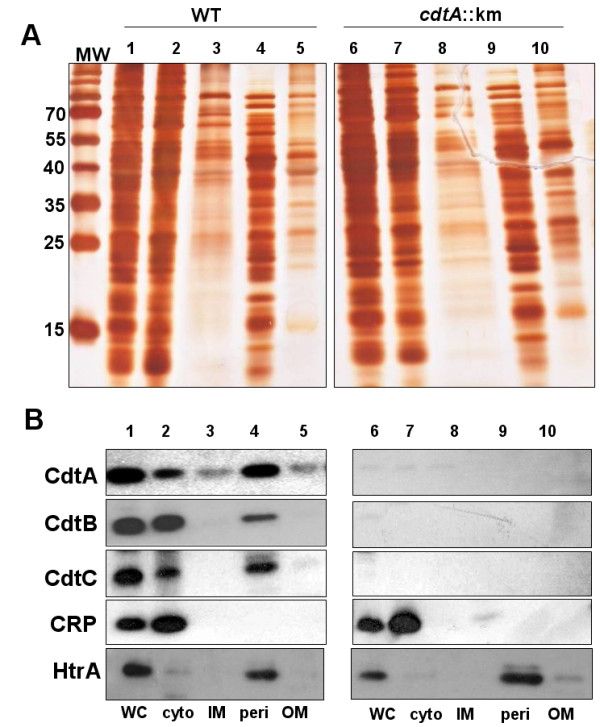
**Analyses of CDT localization in subcellular *C. jejuni *fractions**. Subcellular localization of CDT subunits in *C. jejuni *strain 81-176. (A), SDS-PAGE gel after silver staining and (B), immunoblot analyses of cell fractions from *C. jejuni *wild type strain 81-176 (lanes 1-5) and the *cdtA::km *mutant (lanes 6-10). Lanes 1&6, whole cell lysates; Lanes 2&7, cytoplasmic fractions 3 & 8, inner membrane fractions; Lanes 4 & 9, periplasmic fractions; Lanes 5 & 10, outer membrane fractions. Antisera for detection of CdtA, CdtB, CdtC, CRP, and HtrA, respectively, were used for the immunoblot analyses and representative results of repeated experiments are shown. Molecular weight markers are shown in the lane (MW) on the left. See materials and methods for details about the relative amount of the extracts used.

The presence of CDT in OMVs would imply that the proteins should be localized, at least transiently, in the outer membrane and/or periplasmic compartments of the bacterial cells. We also analyzed the localization of the CDT toxin subunits in different sub-cellular (cytosolic, inner membrane, periplasm, outer membrane) fractions of the bacteria. The results from SDS-PAGE with silver staining (here also serving as a control for protein loading) and immunoblot analysis are shown in Figure 6A&6B, respectively. Antisera directed against the cytosolic marker CRP and the periplasmic protein HtrA was used to further verify the fractionation. All CDT subunits could be detected in the whole cell lysate and in the cytoplasmic fraction (Figure 6B). Some amount of CdtA protein was detected in the membrane factions as well whereas very little of the CtdB and CdtC proteins were detected in those fractions. However, clearly detectable amounts of all CDT proteins were found in the periplasmic fraction (Figure 6B, lane 4). From the relative intensities of the bands detected we could estimate the amount of each Cdt subunit protein in the periplasmic compartment in comparison with that of the cytoplasm. In case of CdtA we estimated that about 50% of the protein appeared in the periplasm whereas only about 5% were detected in the membrane fractions (Figure 6B). The CdtB and CdtC proteins were also present at appreciable levels in the periplasm (about 20% to 30%) in comparison with the levels in the cytoplasm.

From this data, we suggest that substantial amounts of the CDT proteins were translocated into the periplasm of the bacterial cells and from there may be included in the OMVs that are being released from the bacterial cell surface.

### The CDT proteins might be enclosed in the OMVs

**Figure 7:**
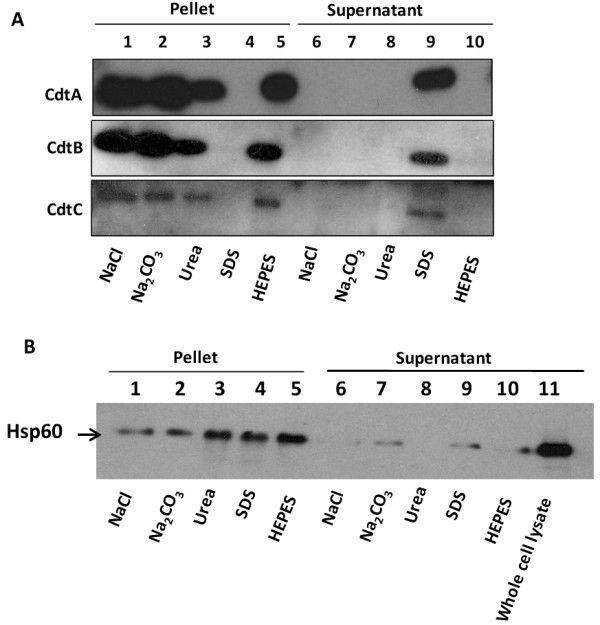
**Analyses of CDT dissociation from OMVs**. (A) Dissociation assays of CDT proteins associated with OMVs from *C. jejuni*. Samples of vesicles in 50 mM HEPES were treated for 60 min on ice in the presence of: NaCl (1 M), Na_2_CO_3 _(0.1 M), urea (8 M) or SDS 1%, respectively. The samples were then centrifuged and the resulting pellets (lanes 1-5) and supernatants (lanes 6-10) were analysed by SDS-PAGE and immunoblot analyses with anti-CdtA, anti-CdtB, and anti-CdtC antisera. (B) Dissociation assays of Hsp60 protein. Samples were treated as in (A), and the immunoblot analysis was done with anti-Hsp60 antiserum.

In order to further assess the nature of the association between CDT proteins and OMVs, we performed a dissociation assay as described in Materials and Methods. As shown in Figure 7A the CDT protein was recovered with OMVs in the pellet after treatment with NaCl, Na_2_CO_3_, Urea, or HEPES buffer pH 7.3. Upon SDS solubilization of the OMVs, however, the CDT proteins could not be detected in the pellet but instead the proteins were released and remained in the supernatant after the subsequent centrifugation (Figure 7A, lanes 4 & 9). These results suggested that CDT was intimately associated with the OMVs. Resistance to high concentration urea (8 M) and liberation after SDS solubilization indicated that the proteins were not merely present as protein aggregates, but were surrounded by a membrane. To verify whether or not the Hsp60 protein was directly associated with OMVs, we monitored its fate in the dissociation assay using the same procedure as was done for CDT proteins. As shown in Figure 7B, the Hsp60 protein was partially released into the supernatant after treatment with Na_2_CO_3 _and SDS but not with Urea. However, most of Hsp60 remained in the pellet even after SDS treatment (Figure 7B, lane 4). Perhaps the formation of protein aggregates after detergent treatment caused Hsp60 to be retained in the pellet. Nevertheless, our results show that CDT and Hsp60 were not associated with OMVs in a similar manner as judged by these assays and the immunoelectron microscopy analysis.

We have also analyzed whether the CDT protein subunits are associated with OMVs in other *C. jejuni *strains. Tests with OMVs samples from the *C. jejuni *strain 81116 by immunoblot analyses using anti-CdtA, anti-CdtB, and anti-CdtC antisera showed clearly that all CDT subunits were associated with OMVs (data not shown). We suggest that the vesicle associated release of CDT proteins is a common feature among *C. jejuni *strains. In this context it is also relevant to mention that a recent proteomic study showed the CDT protein was found to be associated with OMVs derived from the pathogenic *E. coli *strain IHE3034 [44].

### OMV-associated CDT is biologically active

**Figure 8:**
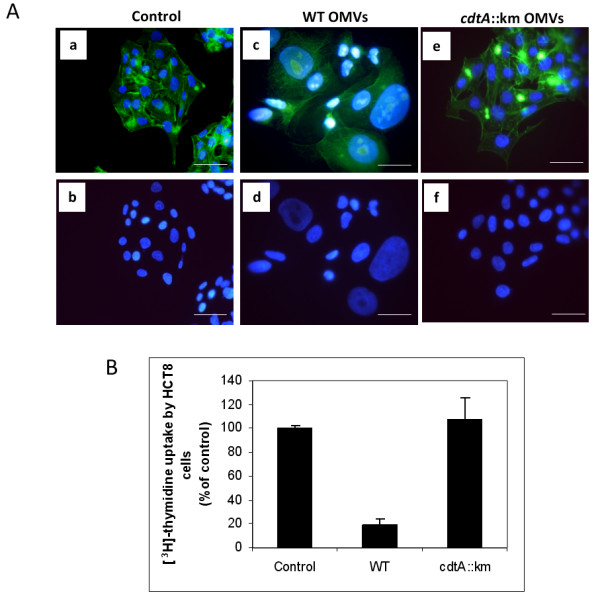
**Analyses of biological activities of CDT**. (A) Cytolethal distending effect by OMVs on HCT8 cells. HCT8 cells without treatment (a, b), HCT8 cells treated with 50 μl of OMVs (total protein concentration was 60 μg/ml) from wild type *C. jejuni *strain 81-176 (c, d), or from the *cdtA*::km mutant (e, f). After 72 hours of treatment the actin filaments and nuclei were stained with phalloidin and DAPI, respectively, as described in materials and methods. Upper panels (a, c, e) show merged images from staining with both dyes and lower panels (b, d, f) show images from DAPI staining only. Bars represent 40 μm. (B) Effect of thymidine uptake on HCT8 cells after treatment with OMVs from wild type *C. jejuni *strain 81-176 and the cdt::km mutant strain DS104 for 48 h. Cells were grown in 96-well plates and 10 μl of OMVs were added to the wells. The results are from triplicate wells and two independent experiments. Data are expressed as mean percentage (± SE).

CDTs constitute a family of genetically related bacterial protein toxins able to stop the proliferation of many different cultured cell lines. The primary effect of the CDTs, regardless of their bacterial origin, is eukaryotic cell cycle arrest at the G2/M stage with resultant cessation of cell division [17]. Since we could detect all CdtA, CdtB, and CdtC subunits in vesicle samples from *C. jejuni *strain 81-176, we decided to test whether the CDT complex was active in such preparations. Earlier studies described that a purified CdtB on its own had no effect on HeLa cells, but when it was combined with CdtA and CdtC the HeLa cells showed cell cycle arrest in the G2/M phase [45]. Results from other studies also indicate that CdtB internalization is necessary for toxicity [46]. In their study, they demonstrated that purified CdtB converts supercoiled plasmid DNA to relaxed and linear forms and promotes cell cycle arrest when combined with an *E. coli *extract containing CdtA and CdtC whereas CdtB alone had no effect on HeLa cells. However introduction of the CdtB polypeptide into HeLa cells by electroporation resulted in cellular distension, chromatin fragmentation, and cell cycle arrest, all of which are consequences of CDT action [46]. In the present study we used a human ileocecum carcinoma cell line (HCT8) instead of the HeLa cell line. We considered that for the analysis of *C. jejuni *infection, a cell line representing the intestinal epithelium might be more relevant. In order to analyze how cultured HCT8 cells were affected by OMVs containing CDT, the cells were treated with the vesicle samples obtained from the *C. jejuni *wild type strain 81-176 and from the *cdtA *mutant strain DS104 (Figure 8A). The CDT-containing vesicle preparations from strain 81-176 induced a distinct enlargement of the HCT8 cells (Figure 8A, panel C&D) that was not observed in case of vesicles from the *cdtA*::km mutant (Figure 8A, panel E&F). As a means to quantify the effect of the OMVs on cell cycle arrest we measured the incorporation of [^3^H]-labeled thymidine by the HCT8 cells that had been treated with OMVs. The thymidine incorporation data clearly indicated that OMVs with CDT caused cell cycle arrest and the level of incorporation was reduced to ca 20% when monitored after 48 h of incubation (Figure 8B).

Taken together, the results in this study demonstrate that biologically active CDT of *C. jejuni *is secreted from the bacteria in association with OMVs. Furthermore, the association of CDT with OMVs was found to be rather tight and we must consider that OMV-mediated release could be a mechanism for delivery of CDT to the surrounding environment and may be involved in the pathogenesis of *Campylobacter *infections.

The present findings are reminiscent of the observations made in case of some toxins and their tight association with OMVs from extra-intestinal pathogenic *E. coli *(ExPEC) but quantitatively there may be noteworthy differences [27,28]. Quantification of the pore forming toxin HlyA, that was secreted and appearing in OMVs from different ExPEC isolates, indicated that it represented a fraction in the range between ca 2%-30%, *i.e*. only a sub-fraction of the exported toxin [28]. Compared with these other cases of toxins exported via OMVs, the present findings are remarkable in that virtually all of the CDT proteins released from the *C. jejuni *cells were found to be OMV-associated

## Conclusion

All CDT subunits from *C. jejuni *were released from the bacterial cells in association with OMVs. The OMV associated toxin caused the cytolethal distending effects on tissue culture cells. Our results strongly suggest that the release of OMV associated CDT is functioning as a route of *C. jejuni *to deliver all the subunits of CDT toxin (CdtA, CdtB, and CdtC) to the surrounding environment, including infected host tissue.

## Abbreviations

CDT: cytolethal distending toxin; OMVs: outer membrane vesicles; LOS: lipooligosaccharide;

## Authors' contributions

BL carried out vesicle isolation, immunoblot analysis, thymidine uptake assay and participated in the study design and drafting of the manuscript. PK carried out vesicle isolation, immunoblot analysis and cytolethal distending assays. YM provided immuno-EM analyses. KV carried out vesicle isolation and immunoblot analysis. TS participated in data analysis. BEU participated in the study design, data interpretation and manuscript writing. PG provided materials and participated in the study design, data interpretation and manuscript writing. SNW had the main responsibility for the study design, data interpretation and manuscript writing. All authors read and approved the final manuscript.
